# Bewegung in der Psychiatrie: Ein Leitfaden für Mediziner:innen in der Patient:innenkommunikation zum Thema körperliche Aktivität, Bewegung und Sport

**DOI:** 10.1007/s40211-025-00535-5

**Published:** 2025-07-29

**Authors:** Carina S. Bichler, Linda K. Rausch, Jana Unterholzner, Martin Kopp, Barbara Sperner-Unterweger, Katharina Hüfner

**Affiliations:** 1 Department für Psychiatrie, Psychotherapie, Psychosomatik und Medizinische Psychologie, Universitätsklinik für Psychiatrie II, Medizinische Universität Innsbruck, Innsbruck, Österreich; 2 Institut für Sportwissenschaft, Universität Innsbruck, Innsbruck, Österreich

**Keywords:** Sportpsychiatrie, Motivierende Gesprächsführung, Bewegungsempfehlungen, Psychische Gesundheit, Sports psychiatry, Motivational interviewing, Physical activity recommendations, Mental health

## Abstract

Psychische Erkrankungen sind häufig mit einem deutlich reduzierten Aktivitätsniveau verbunden, was zu Inaktivitätsraten von 40–86 % führt. Dies ist problematisch, da Bewegung nicht nur positive Auswirkungen auf die körperliche Gesundheit hat, sondern auch eine evidenzbasierte, kostengünstige und risikoarme Behandlungsoption für psychische Störungen darstellt. Dennoch bleibt Bewegung in der psychiatrischen Versorgung unzureichend integriert, was unter anderem auf Barrieren bei Behandelnden und Patient:innen zurückzuführen ist. Zu den größten Hindernissen seitens der Patient:innen zählen Müdigkeit, Unsicherheit und finanzielle Einschränkungen, während Zeitmangel eine geringere Rolle spielt.

Diese Übersicht untersucht die wissenschaftliche Evidenz für Bewegung als Therapiebaustein – „Exercise is Medicine®“ – bei psychiatrischen Erkrankungen und zeigt deren Wirksamkeit für ein breites Spektrum an Indikationen – von „A“ wie Angststörungen bis „Z“ wie Zwangsstörungen. Anschließend wird der „Knowledge-Action-Gap“ zwischen Wissen und Anwendung aufgezeigt und praxisnahe Handlungsempfehlungen aus der Motivierenden Gesprächsführung (Motivational Interviewing) vorgestellt. Neben einer Differenzierung der Begriffe körperliche Aktivität, Bewegung, Sport und Trainingstherapie werden Bewegungsempfehlungen, Techniken zur Verhaltensänderung sowie ein Gesprächsleitfaden und ein Flyer zur Patient:innen-Aufklärung präsentiert.

Ziel des Artikels ist es, Bewegung als festen Bestandteil der psychiatrischen Behandlung zu etablieren und die Anwendbarkeit in der Praxis zu erleichtern. Durch die Integration von Bewegung können nicht nur die Lebensqualität und psychische Gesundheit der Betroffenen verbessert, sondern auch gesundheitliche Disparitäten reduziert werden. Der Artikel bietet evidenzbasierte Empfehlungen und praxisnahe Strategien zur Förderung körperlicher Aktivität in der psychiatrischen Versorgung.

**Zusatzmaterial online:**

Zusätzliche Informationen sind in der Online-Version dieses Artikels (10.1007/s40211-025-00535-5) enthalten.

## Abstract (en)

Mental disorders are often associated with a significantly reduced level of physical activity, leading to inactivity rates of 40–86%. This is concerning, as physical activity not only has well-documented positive effects on physical health but also represents an evidence-based, cost-effective, and low-risk treatment option for psychiatric disorders. However, exercise remains insufficiently integrated into psychiatric care, partly due to barriers among healthcare providers and patients. The most common barriers reported by patients include fatigue, uncertainty, and financial constraints, while lack of time plays a less significant role.

This review examines the scientific evidence supporting exercise as a therapeutic intervention – “Exercise is Medicine®” – for psychiatric disorders and highlights its effectiveness across a wide range of conditions, from anxiety disorders to obsessive-compulsive disorders. Furthermore, it explores the “knowledge-action gap” between existing research and clinical implementation, providing practical recommendations based on Motivational Interviewing techniques. In addition to distinguishing between the concepts of physical activity, exercise, sport, and exercise therapy, this article presents recommendations for physical activity, behavior change techniques, a structured counseling guide, and an informational flyer for patients.

The aim of this article is to establish exercise as an integral component of psychiatric treatment and facilitate its implementation in clinical practice. By integrating physical activity, not only can quality of life and mental health be improved, but health disparities can also be reduced. This article offers evidence-based recommendations and practical strategies for promoting physical activity in psychiatric care.

## Einleitung

Epidemiologische Studien zeigen, dass Menschen mit psychischen Störungen ein geringeres Ausmaß an körperlicher Aktivität aufweisen als die Allgemeinbevölkerung. Die Lebenszeitprävalenz für psychische Störungen wird weltweit auf 29,2 % geschätzt [1]. Dabei beträgt die körperliche Inaktivität im Durchschnitt 31 % für die weltweite, erwachsene Allgemeinbevölkerung und etwa 40–86 % für Menschen mit psychischen Störungen [2]. Der Durchschnittswert der Inaktivität liegt für Personen mit Schizophrenie, bipolarer Depression und Zwangsstörungen bei 55 % [4, 5], während er bei Menschen mit unipolaren Depressionen mit 68 % deutlich höher ausfällt [3].

Mehrere Gründe für die Integration des Therapiebausteins „Bewegung“ in der Behandlung von psychischen Störungen wurden von Fox [6] genannt und sind bis heute aktuell, wie kürzlich von Kopp et al. [7] zusammengefasst:

Die positive Auswirkung von Bewegung auf körperliche Gesundheitsmarker ist umfassend dokumentiert. Da psychische Störungen oft mit einem chronischen Verlauf und körperlichen Problemen einhergehen, wird Bewegung als kostengünstige und nebenwirkungsfreie Behandlungsoption angesehen, welche die körperliche Leistungsfähigkeit und Lebensqualität steigert sowie Morbiditäten und die Mortalität senken kann.

Die weltweit anerkannte Gesundheitsinitiative „Exercise is Medicine® (EIM)“, koordiniert vom American College of Sports Medicine, zielt darauf ab, körperliche Aktivität in Gesundheitsversorgung und Prävention zu integrieren. Aktuell befindet sich das österreichische Zentrum für „EIM®“ als Kooperation zwischen der Universität Innsbruck und der Medizinischen Universität Innsbruck in der Gründungsphase. Das Modellprojekt EIM® Tirol ist Teil der internationalen Gesundheitsinitiative und es werden konkrete Umsetzungsmöglichkeiten mit den regionalen Gegebenheiten am Standort erarbeitet. Dazu gehört unter anderem die Förderung der Gesundheit der österreichischen Bevölkerung durch die Integration von körperlicher Aktivität in die klinische Versorgung und Prävention. Hauptziele sind die routinemäßige Empfehlung von Bewegung, evidenzbasierte Programme zu etablieren und die Zusammenarbeit zwischen Akteur:innen im Gesundheitswesen zu fördern. Eine interdisziplinäre Zusammenarbeit verschiedener Berufsgruppen im rehabilitativen und präventiven Bereich ist essenziell, um „Bewegung als Medikament“ gezielt einzusetzen. Zentrale Rollen übernehmen dabei neben Physiotherapeut:innen die Berufsgruppe der Trainingstherapeut:innen. Trainingstherapeut:innen verfügen über eine fünfjährige universitäre Ausbildung in Sportwissenschaft mit Schwerpunkt Trainingstherapie, die medizinische, trainingswissenschaftliche, psychologische und kommunikationsbezogene Kompetenzen umfasst. Sie arbeiten auf Basis ärztlicher Anordnung vorwiegend in Rehabilitationszentren, Kliniken oder ambulanten Einrichtungen und sind in interdisziplinäre Behandlungsteams eingebunden. Auch bei psychiatrischen Erkrankungen wie Depressionen oder Angststörungen belegen aktuelle Studien die Wirksamkeit trainingstherapeutischer Maßnahmen, weshalb eine systematische Integration dieser Berufsgruppe in der psychiatrischen Versorgung hoch relevant erscheint. Sie fördern gezielt Bewegung und stimmen sich mit anderen Behandelnden ab, um Patient:innen eine gesundheitswirksame Bewegung in Rehabilitation und Prävention zu ermöglichen. Seit 2012 ist die Trainingstherapie gesetzlich verankert. Seit Januar 2025 können Trainingstherapeut:innen auch selbstständig arbeiten. Zudem kann eine ärztliche Überweisung an Trainingstherapeut:innen eine optimale Bewegungsbegleitung sicherstellen.

Ärzt:innen und Personen anderer medizinischer Berufsgruppen wissen häufig sehr gut über die vielschichtigen Vorteile von körperlicher Aktivität Bescheid. Dennoch kann es aus mehreren Gründen schwerfallen, ein Gespräch zum Bewegungsverhalten der:des Patient:in zu führen. Aktuell raten Behandelnde in Schlüsselrollen (z. B. Fachärzt:innen für Psychiatrie, klinische Psycholog:innen, Psychotherapeut:innen) ihren Patient:innen dann zu mehr Bewegung, wenn die Behandelnden selbst körperliche Aktivität als bedeutsam erachten, eine hohe eigene (intrinsische) Motivation für Bewegung haben [8] oder selbst körperlich aktiv sind [9]. Tatsächlich schwieriger als das Aussprechen einer Empfehlung erscheint es Behandelnden, ihre Patient:innen zu mehr körperlicher Aktivität zu motivieren. An dieser Stelle kann die in Kap. 5 beschriebene motivierende Gesprächsführung (Motivational Interviewing) einen wertvollen Beitrag leisten. Durch spezifische Techniken wie empathisches Spiegeln, das Evozieren eigener Veränderungsgründe oder die Entwicklung realistischer Handlungsschritte lassen sich sowohl patientenseitige Barrieren wie Müdigkeit als auch Unsicherheiten auf Seiten der Behandelnden gezielt adressieren.

Die häufigsten Schwierigkeiten von Fachpersonen in der Bewerbung körperlicher Aktivität sind Überzeugungen, dass die Patient:innen Hindernisse zur Durchführung körperlicher Aktivität nicht überwinden könnten – also mangelndes Zutrauen, sowie fehlende Schulung bzw. wenig Übung darin, wie man körperliche Aktivität bei Personen mit psychischen Störungen fördern und unterstützen kann [9]. In der Tat erleben Patient:innen vielfach Hindernisse, die häufigsten sind Müdigkeit, körperliche Einschränkungen und fehlende Fitness, Unsicherheit nach draußen zu gehen, finanzielle Hindernisse und fehlende Begleitung. Der Faktor Zeit stellt umgekehrt bei Menschen mit psychischen Störungen ein signifikant geringeres Hindernis dar als bei Menschen ohne psychische Störung [10].

So finden sich in ambulanten Settings durchaus hohe Abbruchraten bei „verordneten“ Bewegungsprogrammen. Die Dropout-Raten von ambulanten Patient:innen mit gemischten psychischen Störungen liegen hier bei 80 % [11] während in einer Kombination aus Bewegungsprogramm und dem Erlernen von Techniken zur Verhaltensänderung niedrigere Abbruchraten beobachtet wurden [12].

Ziel dieses Artikels ist es, körperliche Aktivität und Bewegung als effektiven Therapiebaustein, zum Beispiel in Form von Trainingstherapie, für die Behandlung psychiatrischer Erkrankungen sichtbar zu machen. Der Artikel intendiert die Auseinandersetzung und nach Möglichkeit die direkte Anwendbarkeit durch seine Leser:innen zum Einsatz in der klinischen Praxis.

**Abb. 1:**
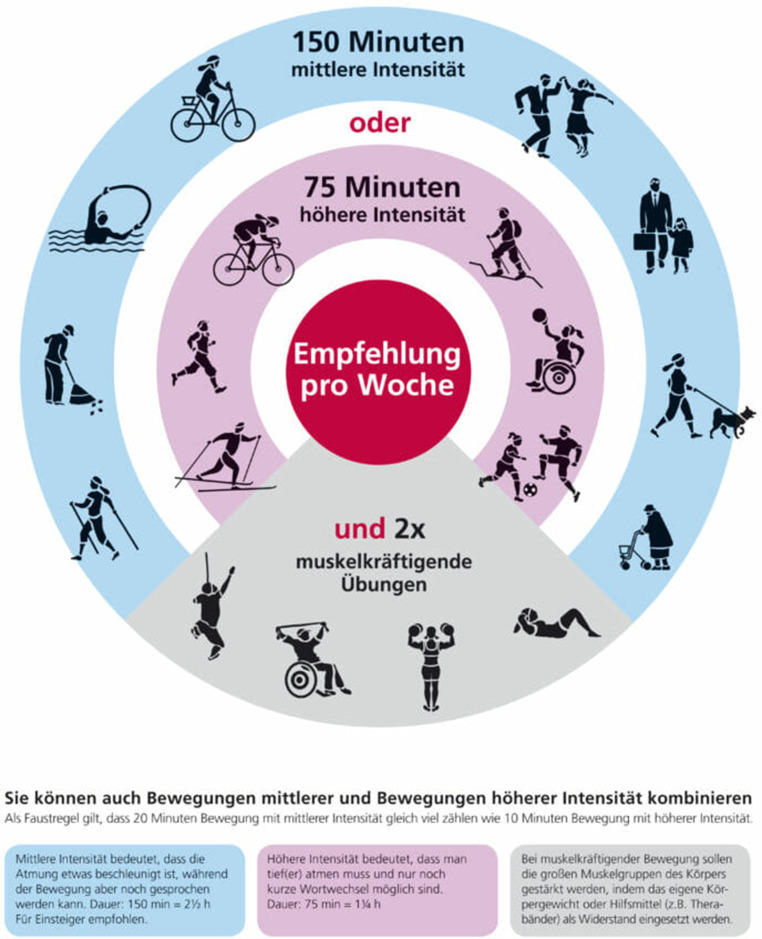
Überblick über aktuelle Bewegungsempfehlungen der Weltgesundheitsorganisation (WHO) bzw. des American College of Sports Medicine (2018). (Mit freundlicher Genehmigung des Fonds Gesundes Österreich)

Der Artikel enthält:

- Begriffsbestimmungen von körperlicher Aktivität, Bewegung und Sport und Trainingstherapie
- Bewegungsempfehlungen (siehe Abb. 1)
- Belege über die Wirksamkeit von körperlicher Aktivität bei Indikationen im psychosomatischen Anwendungsfeld; dazu zählen psychiatrische und häufige komorbide Störungsbilder
- Wissen ≠ Handeln: Möglichkeiten, wie wir dem Knowledge-Action-Gap begegnen können
- Leitfaden für das Gespräch mit Patient:innen
- Flyer für Patient:innen
- Faktencheck „Bewegungsmythen“

## Begriffsbestimmungen

Der Begriff „körperliche Aktivität“ bezeichnet alle Bewegungen der Skelettmuskeln, die mit einem Energieverbrauch – höher als der Grundumsatz – einhergehen [13]. Dazu zählt beispielsweise das Fahrradfahren in die Arbeit, Gartenarbeit oder das Treppensteigen bis zur Wohnungstüre.

„Bewegung“ ist eine Unterkategorie von körperlicher Aktivität, die geplant, strukturiert und wiederholt ist, um eine oder mehrere Komponenten der Fitness zu steigern oder aufrechtzuerhalten [14]. Dazu zählt beispielsweise Joggen, Schwimmen, Krafttraining oder Yoga.

„Sport“ ist eine strukturierte, zielgerichtete, wettbewerbsorientierte, spielerische Form der körperlichen Aktivität [15]. Dazu zählen Sportarten wie beispielsweise Fußball, Leichtathletik, Tennis oder Klettern.

„Trainingstherapie“ bezeichnet einen Behandlungsprozess, der darauf abzielt, durch gezielte und strukturierte körperliche Übungen sowie geeignete Methoden präventiv und rehabilitativ Einfluss auf die Entstehung oder den Verlauf chronischer Erkrankungen zu nehmen [16].

## Bewegungsempfehlungen

(Abb. 1)

## Indikationen und Wirksamkeitsbelege: von „A“ wie Angststörungen bis „Z“ wie Zwangsstörungen

Die Initiative „Exercise is Medicine®“ betont die Bedeutung von Bewegung als integralen Bestandteil der medizinischen Behandlung. Ein gleichnamiger Review-Artikel fasst die wissenschaftliche Evidenz zur Verschreibung von Bewegung als wirksame Therapie zusammen und zeigt deren Nutzen für verschiedene Erkrankungen auf [17]. Im Folgenden werden Krankheitsbilder gelistet, bei denen Bewegung als „Medizin“ präventive und rehabilitative Wirkungsbelege aufweist. Evidenzen werden für die psychiatrischen Erkrankungen Angststörungen, demenzielle Erkrankungen, Depression, Schizophrenie, Suchterkrankungen und Zwangsstörungen angeführt. Im Bereich der neurologischen Erkrankungen werden die Störungsbilder, Multiple Sklerose, Parkinson und Schlaganfall beleuchtet. Die Wirksamkeit der Bewegung auf die häufigsten metabolischen, kardiovaskulären und pulmonalen Erkrankungen und Erkrankungen des Bewegungs- und Stützapparats, sowie onkologische Erkrankungen, werden in Tab. 1 zusammenfassend dargestellt. Ebenso wird auf krankheitsübergreifende Symptome von Schlafstörungen und auf chronischen Schmerzen im Sinne des Bio-Psycho-Sozialen Modells eingegangen.

**Tab. 1:** Beispiele für die Wirksamkeit von Bewegung bei häufigen körperlichen Komorbiditäten psychischer Störungen und somatischen Erkrankungen mit häufigen psychischen Symptomen

	Maßnahmen	Positive Effekte	Referenzen
*Erkrankungen Bewegungsapparat*			
Osteoarthritis	Bewegung allgemein	Schmerzlinderung und funktionale Verbesserung	[32]
Osteoporose	Kraft‑, Ausdauer‑, Gleichgewichtstraining, funktionelles Fitness-Training	Verbesserung der Knochendichte und von allgemeinen gesundheitsbezogenen Variablen	[33, 34]
Rheumatische Arthritis	Ausdauer, Krafttraining, Bewegung im Wasser sowie „Mind-Body-Training“ (Yoga)	Verbesserung der körperlichen Funktionsfähigkeit, Schmerzreduktion	[35]
*Kardiovaskuläre Erkrankungen*			
Arterielle Hypertonie	Isometrische Kraftübungen und kombiniertes Kraft- und Ausdauertraining	Verbesserung des Ruhedrucks und der arteriellen Steifheit	[36, 37]
Claudicatio Intermittens (Schaufensterkrankheit)	Bewegung allgemein	Erhöhung der Geh-Zeit und -Distanz	[38]
Herzinsuffizienz	Strukturierte Bewegungsprogramme	Steigerung der körperlichen Belastbarkeit und der Lebensqualität	[39]
Koronare Herzkrankheit	Bewegungsbasiertes Rehabilitationstraining	Risikoreduktion für kardiovaskuläre Mortalität	[40]
*Metabolische Erkrankungen*			
Adipositas	Kombiniertes Trainings- und Ernährungsprogramm	Langfristige Gewichtsabnahme	[41]
Diabetes Typ 1 und 2	Ausdauer- oder Krafttraining und die Kombination	Steigerung der VO_2Peak_ und Senkung des BMI und des LDL	[42]
Metabolisches Syndrom	Kombination aus Ausdauer- und Krafttraining	Minimierung der kardiovaskulären Risikoparameter	[43]
Polyzystisches Ovarialsyndrom	120 min intensive körperliche Aktivität/Woche, kurze Einheiten	Besserung der kardiorespiratorischen Fitness, Körperzusammensetzung und Senkung der Insulinresistenz	[44, 45]
*Neurologische Erkrankungen*			
Multiple Sklerose	Körperliche Aktivität allgemein	Steigerung der aeroben Kapazität und Muskelkraft, Mobilität und Lebensqualität	[49]
Parkinson-Erkrankung	Körperliche Aktivität allgemein	Steigerung der motorischen Fertigkeit, Gehgeschwindigkeit, -distanz, Alltagsfähigkeiten	[17]
Schlaganfall	Herzkreislauftraining, allgemeine körperliche Aktivität	Verbesserung der allgemeinen Fitness, Mobilität, Gleichgewicht, Gehfähigkeit, -geschwindigkeit, Lebensqualität	[50]
*Onkologische Erkrankungen*			
	Angeleitete Bewegungsprogramme	Verbesserung der Lebensqualität, Reduktion von Ermüdbarkeit sowie Fatigue-Symptome	[51]
*Pulmonale Erkrankungen*			
Asthma bronchiale	Atemtraining, Ausdauertraining, Yoga	Belastbarkeit steigt	[46]
COPD	Anleitung kombiniertes Ausdauer- und Krafttraining	Verbesserte funktionale Kapazität, Leistungsfähigkeit, Lebensqualität und Dysnpnoe	[47]
Mukoviszidose	Mind. 6 Monate Beweggungsintervention	Verbesserung der körperlichen Belastbarkeit	[48]

Alphabetische Reihung der Störungsbilder innerhalb der Kategorien; Auswahl der Krankheitsbilder in Anlehnung an Petersen & Saltin [17]

*VO*_*2Peak*_ maximale Sauerstoffaufnahme, *BMI* Body Mass Index, *LDL* Low Density Lipoprotein

### Angststörungen

Bewegungstherapie zur Behandlung von Angst- und angstassoziierten Störungen wie posttraumatische Belastungsstörung können die Angstsymptomatik signifikant verringern, wobei die Verbesserung meist im kleinen bis mittleren Bereich liegt (geringe bis mittlere Effektstärke) [18]. Bewegung sollte als additiver Therapiebestandteil miteinbezogen werden [19, 20].

### Chronische Schmerzen

Für chronische Rückenschmerzen finden sich Evidenzen für einen signifikant schmerzreduzierenden Effekt bei geringer Effektstärke [21]. Bewegung wirkt möglicherweise auch bei anderen chronischen Schmerzen schmerzlindernd und ist eine Intervention mit wenigen schädlichen Nebenwirkungen, die sich zudem positiv auf die Lebensqualität auswirken kann [22]. Für chronische Muskel- und Skelettschmerzen im Allgemeinen erweist sich insbesondere eine Kombination von Bewegung und Psychoedukation zu psychophysiologischen Zusammenhängen als günstig für die Reduktion von Schmerzen, Beeinträchtigungen, Kinesiophobien und Schmerzkatastrophisierung [23].

### Demenzielle Erkrankungen

Körperliche Aktivität reduziert das Risiko an Alzheimer Demenz und vaskulärer Demenz zu erkranken um durchschnittlich 28 % [24]. Bewegungsprogramme können die kognitive Funktionen bei Personen mit bestehender Demenz signifikant verbessern [19, 25].

### Depression

Bei der Teilnahme an mehrwöchigen Bewegungsprogrammen konnten depressive Symptome mit mittlerer bis großer Effektstärke signifikant reduziert werden. Damit erweist sich Bewegung als Therapeutikum als ebenso wirksam wie pharmakologische, psychotherapeutische und kombinierte Ansätze bei der Reduktion depressiver Symptome [26]. Die größten Effektstärken wurden dabei insbesondere bei Patient:innen mit Major Depression und bei supervidierten Bewegungsinterventionen beobachtet. Aktuelle Übersichtsarbeiten betonen, dass Bewegung vor allem als ergänzende Maßnahme zur Psychotherapie oder medikamentösen Behandlung wirksam ist und so zu einer verbesserten Gesamteffektivität beitragen kann [27]. Empfohlen werden können Yoga, Krafttraining, Gehen/Walken und Jogging [28].

### Schizophrenie

Hier finden sich vielversprechende Auswirkungen von Bewegung mit Evidenzen seit dem Jahr 1981 [17]: Reduktion der Negativ- und Positivsymptomatik, z. B. auditive Halluzinationen, Reduktion von Angst, Depressivität, Distress, Abnahme des Appetits, Lebensqualitätssteigerung, Verbesserung des Kurzzeitgedächtnisses, der Konzentration und Aufmerksamkeit sowie der somatischen Gesundheitsparameter (z. B. Gewicht, Blutfette, Insulin) [19]. Gerade für diese Patient:innengruppe sind die Effekte auf körperliche Komorbiditäten besonders relevant, da ihre Lebenserwartung im Sinne des Mortality-Gaps um 10–25 Jahre verkürzt ist [29].

### Schlafstörungen

Regelmäßige Bewegung, beispielsweise Gehen, Radfahren und Yoga, verbessern die subjektive Schlafqualität und reduzieren die Tagesmüdigkeit und Schwere der Schlafproblematik [30].

### Sucht, Abhängigkeitserkrankung und Craving

Einzelne Trainingseinheiten zeigen sich sehr effektiv in der Reduktion von akutem Substanzcraving. Langzeiteffekte sind noch unzureichend belegt, wobei sich Bewegung positiv auf komorbide Symptome von Depressivität und Ängsten auswirkt und deshalb für diese Patient:innenpopulation gewinnbringend sein kann [7].

### Zwangsstörungen

Bewegung führt zu einer Verringerung der Zwangssymptomatik (große Effektstärke) sowie zu Reduktion von Angst und Depressivität (große Effektstärke), allerdings ist die Studienlage spärlich und mit insgesamt 92 Patient:innen aus sechs Studien gering [31].

## Der Knowledge-Action-Gap

Trotz dieses Wissens über die gesundheitlichen Vorteile von Bewegung bleibt ein großer Teil der Bevölkerung körperlich zu wenig aktiv. Dieses Phänomen, bei dem Wissen nicht in Handeln umgesetzt wird, bezeichnen wir als „Knowledge-Action-Gap“. Diese Lücke zwischen Wissen und Verhalten stellt eine erhebliche Herausforderung für das Gesundheitssystem dar, da insbesondere chronische Erkrankungen häufig mit wiederholtem ungesunden Verhalten verbunden sind. So kann es beispielsweise sein, dass Patient:innen bereits früher schon einmal versucht haben, sich mehr zu bewegen, negative Vorerfahrungen mit Sport gemacht oder ein anfängliches Unbehagen haben. Im Gesundheitswesen zeigt sich dieser Gap, wenn Behandlungsrichtlinien nicht umgesetzt werden, obwohl deren Vorteile bekannt sind. Das Transtheoretische Modell der Verhaltensänderung bietet eine Struktur, um solche Verhaltensänderungen schrittweise zu fördern und beschreibt fünf Phasen:

**Vorüberlegung**: Die Person ist sich der Auswirkungen ihres Verhaltens nicht bewusst.

**Überlegung**: Die Person beginnt, die Auswirkungen ihres Verhaltens zu erkennen.

**Vorbereitung**: Die Person plant konkrete Schritte zur Verhaltensänderung.

**Handlung**: Die Verhaltensänderung wird aktiv umgesetzt.

**Aufrechterhaltung**: Die Person bemüht sich, das neue Verhalten beizubehalten.

Die Motivierende Gesprächsführung ist eine patient:innenzentrierte Methode, die darauf abzielt, die intrinsische Motivation zur Verhaltensänderung zu stärken, indem die Ambivalenz des Gegenübers aufgelöst wird [52]. Dabei steht die Autonomie des/der Patient:in im Vordergrund und es wird auf konfrontative oder überredende Methoden verzichtet.

### Grundsätze der Motivierenden Gesprächsführung

Im Kern der Motivierenden Gesprächsführung steht die Kooperation zwischen Behandler:in und Patient:in, die auf Augenhöhe erfolgt. Dabei geht es nicht darum, den/die Patient:in zu einer Verhaltensänderung zu drängen, sondern vielmehr darum, seine/ihre eigene Motivation und Ressourcen zu aktivieren. Akzeptanz und Wertschätzung spielen eine zentrale Rolle; die Meinungen und Verhaltensweisen des Gegenübers werden respektiert und mit Empathie gewürdigt, unabhängig von unserer eigenen Meinung als Behandler:in. Mitgefühl ist ebenfalls entscheidend, da es darum geht, das Wohl des/der Patient:inn in den Vordergrund zu stellen, ohne eigene Interessen zu verfolgen. Schließlich zielt die Motivierende Gesprächsführung darauf ab, vorhandene Ressourcen und Stärken des Gegenübers zu nutzen (zu evozieren was bereits vorhanden ist), um seine Motivation zur Veränderung zu verstärken, anstatt ihm etwas Neues aufzuzwingen.

### Change Talk

Ein wichtiger Aspekt der Motivierenden Gesprächsführung ist der sogenannte Change Talk, bei dem gezielt Aussagen des/der Patient:in zur Veränderung gefördert werden. Beispielsweise könnte der/die Behandler:in fragen: „Was müsste passieren, damit Sie in Erwägung ziehen, sich regelmäßig zu bewegen?“ oder „Welche Vorteile könnten Sie sehen, wenn Sie diesen Schritt gehen?“ Diese Fragen regen die Person dazu an, über ihre eigenen Gründe für eine Veränderung nachzudenken und diese auszusprechen, was die Wahrscheinlichkeit erhöht, dass die Veränderung tatsächlich umgesetzt wird. Wenn ein:e Patient:in beispielsweise sagt: „Ich denke, es wäre gut für meine Gesundheit, wenn ich Sport mache,“ könnte der/die Behandler:in antworten: „Was hält Sie davon ab, diesen Schritt jetzt zu gehen?“ Dadurch wird die Motivation zur Veränderung weiter gestärkt. Eine weitere Möglichkeit ist die Frage nach der eigenen Einschätzung der Veränderungsmotivation auf einer Skala, gefolgt von der Nachfrage, warum die Bewertung nicht niedriger ausfällt, um positive Aspekte zu verstärken. Die behandelnde Person fragt: „Wie stark fühlen Sie sich auf einer Skala von 0 bis 10 motiviert, Ihr Verhalten zu ändern? Null ist dabei keine und 10 die höchste Motivation“ Wenn der/die Patient:in antwortet: „Vielleicht so um die 5,“ könnte der nächste Schritt sein, zu fragen: „Wieso eine 5 und keine 3?“ Diese Frage hilft dem/der Patient:in, die positiven Aspekte und die Gründe für eine Veränderung zu reflektieren und zu artikulieren. Indem eine niedrigere Zahl als die genannte in den Raum gestellt wird, wird das Gegenüber dazu angeregt, über seine vorhandene Motivation nachzudenken und diese weiter zu verstärken. Sollte die Person jedoch eine sehr niedrige Zahl wie 0 nennen, deutet dies darauf hin, dass sie sich noch nicht im Zustand der Ambivalenz befindet, und es wäre notwendig, früher im Veränderungsprozess anzusetzen.

## Leitfaden für das Gespräch mit Patient:innen

Der Gesprächsleitfaden wurde anhand konzeptueller Inhalte der motivierenden Gesprächsführung [53] erstellt und richtet sich an klinisch tätige Fachkräfte, z. B. Ärzt:innen, Psycholog:innen, Psycho‑, Physio‑, Ergo- und Trainingstherapeut:innen; er lässt sich je nach Bedarf im direkten Patient:innenkontakt sowohl vollständig als auch punktuell einsetzen. Erste Erfahrungen durch psychiatrisches Fachpersonal liegen bereits vor. Dieser Leitfaden soll einen niederschwelligen Zugang zur Thematik eröffnen und umfasst zehn Schritte zur nachhaltigen Thematisierung des Bewegungsverhaltens von Patient:innen. Enthalten sind thematisch aufbauende Themenschritte, beginnend mit einer Anamnese des aktuellen Bewegungsverhaltens. Es folgen Informationsvermittlung und Psychoedukation sowie die gemeinsame Erarbeitung einer individuellen Zielsetzung, bei welcher der SMART-Zielrahmen (Spezifisch, Messbar, Erreichbar, Relevant, Zeitgebunden) [54] als hilfreiche Orientierung dienen kann, um realistische und motivierende Ziele zu formulieren. Durch das Setzen klarer, messbarer, realistisch erreichbarer und zeitlich einschätzbarer Ziele kann die Motivation zur Verhaltensänderung wesentlich erhöht werden. Daran anschließend erfolgt die Entwicklung eines Aktivitätsplans und antizipatorische Auseinandersetzung mit möglichen individuellen Hindernissen bis hin zu den bereits bekannten, häufigen motivationalen Barrieren. Abschließend wird eine Struktur zur weiteren Planungs- und Zielüberprüfungsschritten dargeboten. Im Leitfanden finden sich konkrete Formulierungsbeispiele in kursiver Schrift und es wird die Möglichkeit geboten, den Bogen direkt im Gespräch mit einer/m Patient:in auszufüllen; entsprechende Felder zum Ausfüllen sind in den Kästen vorgesehen. Der Gesprächsleitfaden findet sich in Anhang A.

## Flyer für Patient:innen

Ein Flyer zur Weitergabe an Patient:innen befindet sich in Anhang B. Auf dem Flyer wurden Anregungen und praktische Tipps zur Unterstützung einer Verhaltensänderung hinsichtlich mehr körperlicher Aktivität aus wissenschaftlicher Evidenz abgeleitet und zusammengefasst.

## Faktencheck „Bewegungsmythen“

Im Folgenden werden sechs Mythen dargestellt und mit wissenschaftlichen Belegen diskutiert. Die dargestellten Mythen beschränkt sich auf eine kleine Auswahl jener Fehlinformation, mit der Patient:innen im psychiatrischen Kontext möglicherweise an Behandler:innen herantreten. Eine kurze und evidenzbasierte Erläuterung soll eine patient:innengerechte Auseinandersetzung mit den möglichen Fehlinformationen ermöglichen.

### „Schmerz als guter Ratgeber“

Eine Sichtweise, die auf akuten Schmerzen oder Traumata basiert, prägte lange Zeit das Management von muskuloskeletalen Schmerzen. Während die Einstellung „wenn es wehtut, lass es bleiben“ bei traumatischen Verletzungen wirksam ist, führt sie bei chronischen und rezidivierenden Schmerzen, wie sie beispielsweise häufig bei Personen mit somatoformen Störungen zu finden sind, zu einem Rückgang der körperlichen Leistungsfähigkeit. Das Motto „Schmerz als Ratgeber“ ist also nach einer Verletzung wie beispielsweise einer Bänderverletzung oder einem Bruch sinnvoll. Bei wiederkehrendem oder chronischem Schmerz ist eine allmähliche Reaktivierung in einem supervidierten Rahmen ratsam, da Schmerz nicht immer ein guter Wegweiser ist und zu Aktivitätsvermeidung führen kann [55].

### „No pain no gain“

Sportler:innen erkennen häufig den Unterschied zwischen Schmerzen aufgrund einer Verletzung und solchen durch intensives Training. Die Trennlinie zwischen einem erfolgreichen Training und Überanstrengung ist manchmal sehr fein, und dennoch entscheidend für erfolgreiches Schmerzmanagement, Rehabilitation und Leistungssteigerung [55]. Der Begriff „Pacing“ beschreibt ein Therapiekonzept, welches Patient:innen beispielsweise im Rahmen der Behandlung von Fatigue oder einer Long-Covid-Symptomatik vermittelt wird. „Pacing“ bedeutet eine Balance zwischen Schonung und Aktivierung zu finden [56]. Der Grundsatz „No pain no gain“ bzw. „Ohne Schmerz kein Gewinn“ ist als therapeutischer Grundsatz somit ebenfalls unangebracht und kann zu Überanstrengung und folglich zu einem Aktivitätsabbruch führen.

### „Weniger als 150 min Sport pro Woche bringt nichts“

Die Weltgesundheitsorganisation empfiehlt in ihrer Leitlinie zu körperlicher Aktivität ein Minimum von 150 Minuten moderater Ausdaueraktivität pro Woche sowie zusätzlich muskelkräftigende Übungen an mindestens zwei Tagen. Studienergebnisse konnten sowohl zeigen, dass ein höheres Ausmaß an körperlicher Aktivität mehr gesundheitsförderliche Effekte hervorbringt [57], als auch, dass bereits weniger als die empfohlenen 150 Minuten einen positiven Gesundheitsbeitrag leisten [58]. Gerade für Personen mit geringer Selbstwirksamkeitserwartung – ein häufiges Merkmal bei depressiven und Angststörungen – kann es motivierend sein zu wissen, dass bereits kleine Schritte einen Unterschied machen. In diesem Zusammenhang wird vom sogenannten *Bottom-up-Effekt* gesprochen: Menschen mit sehr niedrigem Ausgangsniveau an Bewegung profitieren oftmals überproportional stark von ersten Aktivitäten [59]. Dies legt nahe, dass gesundheitliche Effekte durch körperliche Aktivität entlang eines Kontinuums zunehmen – und dass der Einstieg wichtiger ist als Perfektion. Gleichzeitig gilt es, auch Übermaß zu vermeiden: Sehr hohe Aktivitätslevel zeigen in Studien keine lineare Zunahme positiver Effekte und können im Extremfall mit gesundheitlichen Nachteilen verbunden sein. Dies führt zur kritischen Betrachtung des nächsten verbreiteten Irrtums:

### „Je mehr Sport, desto besser“

Ein Großteil der Patient:innen läuft keine Gefahr, sich aufgrund zu hoher körperlicher Aktivitätsraten selbst körperlich oder psychisch zu schaden. So führte beispielsweise die vierfache „Dosis“ des empfohlenen Mindestmaßes von körperlicher Aktivität zu keinen gesundheitsschädigenden Auswirkungen [57]. Dennoch ist im Bereich der stoffungebundenen Suchterkrankungen darauf hinzuweisen, dass ein sehr hohes Bewegungsausmaß auch in eine „Sportsucht“ führen kann. Weder im ICD-10 noch im ICD-11 noch im DSM‑5 wird die „Sportsucht“ also solche angeführt. Die klassischen Kriterien für eine Abhängigkeitserkrankung können auch bei einem zu hohen Ausmaß an Sport angelegt werden, darunter Dosissteigerung, Einschränkung in wichtigen Lebensbereichen und Beibehalten des Suchtverhaltens trotz negativer gesundheitlicher Konsequenzen, beispielsweise nicht-Einhaltung von Verletzungspausen und Ermüdungsbrüche [60].

### „Kein Sport in der Schwangerschaft“

Schwangerschaft stellt keine Kontraindikation für kontrollierte Bewegung und Sport dar. Regelmäßige körperliche Aktivität während der Schwangerschaft ist nicht nur vorteilhaft, sondern auch essentiell für die physische und psychische Gesundheit der Mutter. Körperliche Aktivität kann die Bewältigungsfähigkeiten während der Geburt und das Erreichen von Fitnesszielen nach der Geburt unterstützen. Zusätzlich zu dem allgemeinen Nutzen bieten sich während der Schwangerschaft spezifische gesundheitliche Vorteile, darunter die Prävention von Schwangerschaftsdiabetes, die Reduktion des Präeklampsie-Risikos, eine höhere Wahrscheinlichkeit der Geburt eines normalgewichtigen Babys und die Vermeidung von übermäßiger Gewichtszunahme der Mutter. Regelmäßige Bewegung kann Symptome postpartaler Depression reduzieren [61]. Gerade bei Patientinnen mit psychischen Vorerkrankungen können jedoch Unsicherheiten oder irrationale Überzeugungen wie „Bewegung in der Schwangerschaft ist gefährlich“ oder „Ich belaste damit mein ungeborenes Kind“ auftreten. In diesem Zusammenhang ist es besonders hilfreich, solche Mythen im Sinne eines zielgruppenspezifischen Faktenchecks zu entkräften und gezielt auf häufige Sorgen einzugehen.

### „Nur bei moderatem Training nimmt man ab“

Diesem Mythos liegt eine Verwechslung zwischen Fettstoffwechsel und Kalorienverbrauch zugrunde. Zwar wird im moderaten Intensitätsbereich prozentual mehr Energie aus Fetten gewonnen als bei hoher Intensität, dennoch ist der absolute Kalorienverbrauch hier meist geringer. Der Fettstoffwechsel liefert etwa die Hälfte der Energie im Vergleich zum Kohlenhydratstoffwechsel, der besonders bei intensiveren Belastungen aktiviert wird, wenn der Körper schnell verfügbare Energie benötigt [62]. Bei moderaten Aktivitäten greift der Körper vermehrt auf den effizienteren Fettstoffwechsel zurück, um über längere Zeit Energie bereitzustellen. Entscheidend für eine Gewichtsreduktion ist jedoch die Gesamtbilanz des Kalorienverbrauchs: Eine Stunde hochintensives Intervalltraining führt zu einem deutlich höheren Energieverbrauch als eine Stunde lockeres Joggen. Empfehlungen für Patient:innen mit dem Ziel der Gewichtsreduktion, sollten daher auf eine Steigerung des gesamten Kalorienverbrauchs ausgerichtet sein. Bewegungsformen wie Nordic Walking oder zügiges Gehen mit Stöcken können durch den Einsatz der oberen Extremitäten und die Aktivierung der Rumpfmuskulatur den Energieverbrauch zusätzlich erhöhen. Gleichzeitig ermöglichen sie ein höheres Gehtempo – bei geringem Risiko für Überlastung [63].

## Vertiefende Literatur

- Die **Motivierende Gesprächsführung** ist eine etablierte Methode mit nachgewiesener Wirksamkeit zur Förderung der Veränderungsmotivation bei verschiedenen verhaltensbedingten Gesundheitsproblemen. Praktische Anwendungsbeispiele finden sich im Standardwerk von Miller & Rollnick [52].
  Eine evidenzbasierte Darstellung für die ärztliche Praxis bietet zudem der Artikel im *Deutschen Ärzteblatt*: [64]: https://www.aerzteblatt.de/archiv/217808/Motivierende-Gespraechsfuehrung-Ein-evidenzbasierter-Ansatz-fuer-die-aerztliche-Praxis
- Die Kommunikation über **Übergewicht und Adipositas** stellt sowohl im klinischen als auch im privaten Kontext häufig eine Herausforderung dar. Eine aktuelle und praxisnahe Übersichtsarbeit im *Journal of the American Medical Association (JAMA)* bietet evidenzbasierte Empfehlungen zur Patient:innen-Arzt-Kommunikation bei Gewichtsmanagement und Lebensstiländerungen [65]: Patient-Clinician Communication About Weight Loss | Lifestyle Behaviors | JAMA | JAMA Network
- **Bewegungsempfehlungen** für verschiedene Altersgruppen und Lebenssituationen, herausgegeben vom Fonds Gesundes Österreich, sind detailliert unter folgendem Link verfügbar:
https://fgoe.org/sites/fgoe.org/files/2022-01/WB_17_bewegungsempfehlungen_bfrei.pdf

## Supplementary Information

Anhang A: Gesprächsleitfaden

Anhang B: Flyer für Patient:innen

## Data Availability

In dieser Übersichtsarbeit wurden alle verwendeten Quellen vollständig angegeben und referenziert. Darüber hinaus sind Links und Verweise zu weiterführender Literatur enthalten. Der Gesprächsleitfaden (Anhang A) sowie der Flyer zur Patient:inneninformation (Anhang B) stehen für die klinische Nutzung frei zur Verfügung. Für eine darüber hinausgehende bzw. kommerzielle Nutzung ist das Einverständnis der Autor:innen einzuholen.
