# Supplementary material for: Physical activity in psychiatry: a practical guide for clinicians on patient communication about physical activity, exercise, and sport
Source: Neuropsychiatr. 2025 Jul 29;39(3):133–43. [Article in German] doi: 10.1007/s40211-025-00535-5 (PMC12396993; doi:10.1007/s40211-025-00535-5)
Supplement: Supplementary file 1 — Anhang A: Gesprächsleitfaden [file 40211_2025_535_MOESM1_ESM.docx]

## Anhang A: Gesprächsleitfaden

Gesprächsleitfaden für Ärzt:innen, Psycholog:innen, Psycho-, Physio-, Ergo- und Trainingstherapeut:innen und andere klinisch tätige Berufsgruppen zur Förderung der körperlichen Aktivität bei Patient:innen**^[[1]](#footnote-1)^**

Name Patient:in: ___________________________________ Geburtsdatum: ___________

Durchführungsdatum: __________________

Einführung:

- Schaffen Sie eine angenehme Gesprächsatmosphäre und stellen Sie sicher, dass Sie ein Zeitfenster von mindestens 15~~0~~ Minuten eröffnen können.
- Erklären Sie, warum Sie heute über das Thema körperliche Aktivität sprechen möchten, zum Beispiel:
  *Vielen Patient:innen mit Angststörungen* (zutreffende Diagnose verwenden) *erleben Bewegung als eine hilfreiche Möglichkeit im Umgang mit der Angst, da Bewegung die Stressregulierung unterstützt und Angst reduzieren kann.*

**1. Patient:innenhistorie und Aktivitätsniveau verstehen:**

- Eröffnen Sie die Thematik, indem Sie nach der aktuellen körperlichen Aktivität des/r Patient:in fragen.
  *Wenn Sie an eine typische Woche denken, wie viel bewegen Sie sich?*
- Erfragen Sie eventuelle gesundheitliche Probleme oder körperliche Einschränkungen, welche die körperliche Aktivität beeinflussen könnten.

Aktivität Dauer Anzahl pro Woche

_____________________________________________________________________________________________________________________________________________________________________________________________________________________________________________________________________

Einschränkungen und Probleme:
______________________________________________________________________________________________________________________________________________________________

*Gibt es gesundheitliche Probleme oder Schmerzen, die Ihre Bewegung einschränken?*

**2. Wissensvermittlung:**

- Erklären Sie die gesundheitlichen Vorteile regelmäßiger körperlicher Aktivität.
  Lassen Sie mich kurz erläutern, wie regelmäßige Bewegung Ihre Gesundheit positiv beeinflussen kann. *Haben Sie schon einmal von den Vorteilen von Bewegung für …* (zutreffende Beschwerden der/s Patient:in nennen) *die Stressregulation, das Herz, die Stimmung, etc. gehört?*
- Betonen Sie die Bedeutung der Prävention von Krankheiten und gesundheitlichen Problemen durch Bewegung.

**3. Individuelle Ziele setzen:**

- Erheben Sie zunächst die Bereitschaft zur körperlichen Aktivität und klären Sie, ob die Patient:innen bereits Erfahrungen oder Überlegungen dazu angestellt haben.
- Bitten Sie den/die Patient:in, realistische, verhaltensbezogene Ziele für die eigene körperliche Aktivität (z.B. 3x abends nach dem Abendessen für 30 Minuten spazieren gehen) festzulegen. Diese sollten den aktuellen Gesundheitszustand und die persönlichen Präferenzen berücksichtigen.
- *Welche Ziele würden Sie gerne in Bezug auf Bewegung erreichen? Wann und wie könnten Sie Bewegung machen?*
- Helfen Sie dabei, klare, messbare und zeitlich begrenzte Ziele zu formulieren, um die Motivation zu steigern.

Ziele der/des Patient:in:

_____________________________________________________________________________________________________________________________________________________________________________________________________________________________________________

**4. Aktivitätsplan entwickeln:**

- Besprechen Sie verschiedene Arten von Aktivitäten, die für die/den Patient:in geeignet sein könnten, basierend auf den Zielen und Einschränkungen. Ermutigen Sie die/den Patient:in, Freude an der Bewegung zu finden, indem Sie Aktivitäten auswählen, bei der bereits eine Affinität besteht oder welche der Person bereits früher Freude bereitet haben.

*Lassen Sie uns gemeinsam einen Aktivitätsplan erstellen, der zu Ihnen passt. Welche Aktivitäten interessieren Sie, und wie könnten Sie diese in Ihren Alltag integrieren?*

- Erarbeiten Sie gemeinsam einen Aktivitätsplan, der Art, Intensität, Dauer und Häufigkeit der Übungen enthält.

Aktivitätsplan:

Aktivität Wann? Wie lange? Anmerkungen

____________________________________________________________________________________________________________________________________________________________________________________________________________________________________________________________________________________________________________________________

**5. Barrieren und Lösungen besprechen:**

- Erfragen Sie mögliche Hindernisse, welche den/die Patient:in davon abhalten könnten, aktiv zu sein, z. B. Zeitmangel, Schmerzen, fehlende Trainingspartner:in, Ausrüstung/finanzielle Einschränkungen oder fehlende Motivation.

*Gibt es Hindernisse oder Bedenken, die Sie daran hindern könnten, aktiver zu sein? Zum Beispiel Zeitmangel oder körperliche Beschwerden?*

- Gemeinsam erarbeiten Sie Strategien, um diese Barrieren zu überwinden. Geben Sie praktische ~~Ratschläge~~ Empfehlungen und Tipps – zum Beispiel, Aktivitäten mit geringerer Intensität wie Yoga oder gemütliches Walken auszuprobieren oder Bewegung in kürzere Einheiten aufzuteilen, wenn Müdigkeit ein Hindernis darstellt. Zögert die Person etwa, das Haus zu verlassen, kann Bewegung gemeinsam mit einer vertrauten Person oder in einer belebten bzw. als sicher empfundenen Umgebung hilfreich sein.

Hindernisse Was dagegen unternommen wird

___________________________________________________________________________________________________________________________________________________________________________________________________________________________________________________________________________________________________________________________________________________________________________________________________________

**6. Sicherheit und Gesundheitsbedenken klären:**

- Sprechen Sie über die Bedeutung des richtigen Aufwärmens, Dehnens und einer angemessenen Trainingsintensität, um Verletzungen zu vermeiden.

*Wir sollten sicherstellen, dass Sie Ihre Aktivitäten sicher ausführen. Denken Sie daran, sich vorher aufzuwärmen und achtsam auf Ihren Körper zu hören. Falls nötig, können wir auch weitere medizinische Untersuchungen durchführen*.

**7. Motivation fördern:**

- Ermutigen Sie die/den Patient:in, ein Aktivitätstagebuch zu führen, um Fortschritte zu verfolgen und motiviert zu bleiben.

*Haben Sie schon einmal überlegt, ein Aktivitätstagebuch zu führen? Das kann Ihnen helfen, Ihre Fortschritte zu verfolgen und Ihre Motivation aufrechtzuerhalten.*

- Besprechen Sie die Möglichkeit, eine:n Trainingspartner:in zu finden oder sich einer Sportgruppe anzuschließen, um soziale Unterstützung zu erhalten.

*Auch die Unterstützung von Freunden oder eine Sportgruppe kann hilfreich sein.*

**8. Folgegespräch und Unterstützung anbieten:**

- Vereinbaren Sie einen Termin für ein weiteres Gespräch, um den Fortschritt der/s Patient:in zu überprüfen und Anpassungen am Aktivitätsplan vorzunehmen.

*Lassen Sie uns einen Termin für unser nächstes Treffen vereinbaren, damit Sie Ihre Ziele gut verfolgen und möglicherweise auftretenden Schwierigkeiten besser begegnen können.*

- Geben Sie Informationen über Ressourcen wie Fitnesskurse, Sportvereine oder Online-Programme, die der/dem Patient:in bei der Umsetzung seines Aktivitätsplans helfen können. Flyer oder Ausdrucke bestehender Angebote können hilfreich sein.

**9. Zusammenfassung und Abschluss:**

- Fassen Sie die wichtigsten Punkte des Gesprächs zusammen und ermutigen Sie die/den Patient:in, Fragen zu stellen.

*Um zusammenzufassen, haben wir heute darüber gesprochen, wie Sie Ihre körperliche Aktivität steigern können. Sie haben sich das Ziel gesetzt, dass Sie …*

- Schreiben Sie den Aktivitätsplan auf bzw. lassen die/den Patient:in diesen aufschreiben und mitnehmen
- Erinnern Sie die/den Patient:in daran, dass Sie für weitere Unterstützung und Beratung zur Verfügung stehen und stellen Sie ggf. sicher, dass der/die Patient:in nicht unter Druck gerät.

*Sie sollten wissen, dass es gar nicht so einfach ist, Gewohnheiten zu verändern. Manchmal brauchen Veränderungen Zeit und Rückschläge sind ganz normal. Bitte üben Sie Nachsicht mit sich selbst, wenn es nicht gleich auf Anhieb so klappt wie Sie sich das vorstellen.*

**10. Dokumentation:**

- Protokollieren Sie das Gespräch und die festgelegten Ziele in der Patient:innenakte, um die Verfolgung des Fortschritts zu erleichtern. Die vereinbarten Ziele sollen im Folgegespräch wieder besprochen und ggf. angepasst werden.

Einschätzung des Grads der Motivation für körperliche Aktivität und Verhaltensänderung

Keine mäßig hoch

1. Im Folgenden wird eine Gesprächsmöglichkeit mit Formulierungsvorschlägen (in kursiv gehalten) und Ausfüllboxen für besprochene Inhalte (in grauem Kasten) beschrieben. Die Formulierungsvorschläge sollten nicht als Einschränkung der fachlichen Kompetenz in der direkten Gesprächsinteraktion mit der/m Patient:in verstanden werden und verfolgen lediglich das Ziel der praktischen Anschaulichkeit. Der Leitfaden erhebt keinen Anspruch auf Vollständigkeit. [↑](#footnote-ref-1)
