# Supplementary figures and images for: Physical activity in psychiatry: a practical guide for clinicians on patient communication about physical activity, exercise, and sport
Source: Neuropsychiatr. 2025 Jul 29;39(3):133–43. [Article in German] doi: 10.1007/s40211-025-00535-5 (PMC12396993; doi:10.1007/s40211-025-00535-5)

## Anhang B: Flyer für Patient:innen


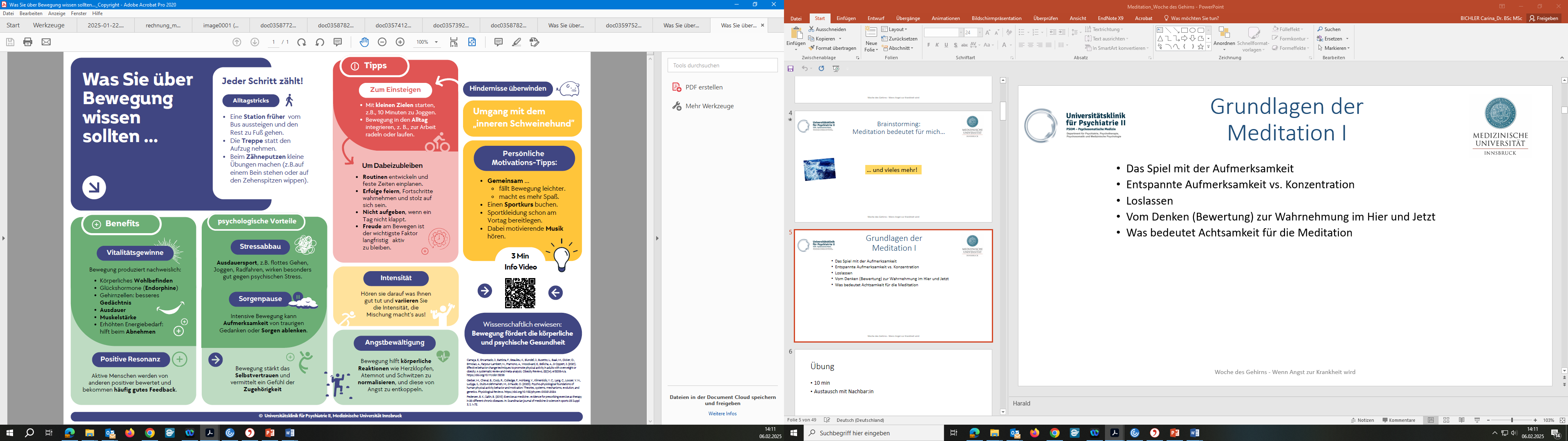

Supplement: Supplementary file 2 — Anhang B: Flyer für Patient:innen [file 40211_2025_535_MOESM2_ESM.docx]
